# In the Absence of Effects: An Individual Patient Data Meta-Analysis of Non-response and Its Predictors in Internet-Based Cognitive Behavior Therapy

**DOI:** 10.3389/fpsyg.2019.00589

**Published:** 2019-03-29

**Authors:** Alexander Rozental, Gerhard Andersson, Per Carlbring

**Affiliations:** ^1^Department of Clinical Neuroscience, Karolinska Institutet, Stockholm, Sweden; ^2^Institute of Child Health, University College London, London, United Kingdom; ^3^Department of Behavioural Sciences and Learning, Linköping University, Linköping, Sweden; ^4^Department of Psychology, Stockholm University, Stockholm, Sweden; ^5^Department of Psychology, University of Southern Denmark, Odense, Denmark

**Keywords:** negative effects, non-response, predictors, individual patient data meta-analysis, Internet-based cognitive behavior therapy

## Abstract

**Background:** Negative effects of psychological treatments have recently received increased attention in both research and clinical practice. Most investigations have focused on determining the occurrence and characteristics of deterioration and other adverse and unwanted events, such as interpersonal issues, indicating that patients quite frequently experience such incidents in treatment. However, non-response is also negative if it might have prolonged an ongoing condition and caused unnecessary suffering. Yet few attempts have been made to directly explore non-response in psychological treatment or its plausible causes. Internet-based cognitive behavior therapy (ICBT) has been found effective for a number of diagnoses but has not yet been systematically explored with regard to those patients who do not respond.

**Methods:** The current study collected and aggregated data from 2,866 patients in 29 clinical randomized trials of ICBT for three categories of diagnoses: anxiety disorders, depression, and other (erectile dysfunction, relationship problems, and gambling disorder). Raw scores from each patient variable were used in an individual patient data meta-analysis to determine the rate of non-response on the primary outcome measure for each clinical trial, while its potential predictors were examined using binomial logistic regression. The reliable change index (RCI) was used to classify patients as non-responders.

**Results:** Of the 2,118 patients receiving treatment, and when applying a RCI of *z* ≥ 1.96, 567 (26.8%) were classified as non-responders. In terms of predictors, patients with higher symptom severity on the primary outcome measure at baseline, Odds Ratio (*OR*) = 2.04, having a primary anxiety disorder (*OR* = 5.75), and being of male gender (*OR* = 1.80), might have higher odds of not responding to treatment.

**Conclusion:** Non-response seems to occur among approximately a quarter of all patients in ICBT, with predictors related to greater symptoms, anxiety disorders, and gender indicating increasing the odds of not responding. However, the results need to be replicated before establishing their clinical relevance, and the use of the RCI as a way of determining non-response needs to be validated by other means, such as by interviewing patients classified as non-responders.

## Introduction

Negative effects of psychological treatments are a relatively unchartered territory in both research and clinical practice. Despite being recognized early in the scientific literature (c.f., Strupp and Hadley, 1977), empirical evidence for their occurrence and characteristics have been quite scarce, but has currently received increased attention (Rozental et al., 2018). Bergin (1966) provided the first report of the “client-deterioration phenomenon” (p. 236), referred to as the deterioration effect, i.e., patients faring worse in treatment. Since then, several studies have investigated the rate of worsening in different naturalistic settings (c.f., Hansen et al., 2002; Mechler and Holmqvist, 2016; Delgadillo et al., 2018), while a number of systematic reviews have assessed deterioration among patients in randomized controlled trials (c.f., Ebert et al., 2016; Rozental et al., 2017; Cuijpers et al., 2018), estimating that ~5–10% of those in treatment for depression and anxiety disorders deteriorate. In comparison to a wait-list control, the odds ratio for deterioration in treatment is nevertheless lower, suggesting that the benefits of receiving help still outweigh the risks (Karyotaki et al., 2018). Recent attempts to identify variables related to worsening have also revealed that sociodemographics variables like lower educational level are linked to increased odds of deterioration (Ebert et al., 2016), while older age and having a relationship constitute protective factors (Rozental et al., 2017). This implies that certain features might be important to consider in relation to treatment, although more research is needed to determine if and how this could be clinically useful.

Meanwhile, others have stressed the importance of monitoring the potential adverse and unwanted events that may occur in treatment, which are not necessarily related to symptoms (Mays and Franks, 1980). This can include interpersonal issues, stigma, and feelings of failure, identified using therapist checklists (Linden, 2013), self-reports completed by patients (Rozental et al., 2016), or open-ended questions (Rozental et al., 2015). Such incidents have been even less explored, although a few recent attempts have found that almost half of the patients are experiencing negative effects at some time in treatment (Rheker et al., 2017; Moritz et al., 2018; Rozental et al., inpress). Whether or not these are in fact detrimental is an issue that warrants further investigation. Rozental et al. (2018) argued that even though adverse and unwanted events seem to exist, it is still unclear if they affect treatment outcome. Some might even be regarded as a necessary evil, such as temporary bouts of increased anxiety during exposure exercises in Cognitive Behavior Therapy (CBT). In addition, there is also an ongoing debate on how to define and measure adverse and unwanted events occurring in treatment, with different taxonomies having been proposed, which makes it difficult to systematically assess and report such incidents across studies (Rozental et al., 2016).

While most of the scientific literature on negative effects deal with the issue of inflicting something on the patient, e.g., novel symptoms and deterioration, less thought has been given to the *absence* of effects. Dimidjian and Hollon (2010) were early to raise the problem with non-response in treatment, arguing that no improvement at all could potentially have restricted the patient from accessing a more effective treatment. From this perspective, a treatment without any benefits would also be seen as negative given that it may have prolonged an ongoing condition and caused unnecessary suffering, and that “it still may be costly in terms of time, expense, and other resources” (p. 24). However, they also pointed out that this has to be put in relation to the natural course of the psychiatric disorder for which one has been treated, which complicates the issue of classifying non-response. Linden (2013) defined non-response as “Lack of improvement in spite of treatment” (p. 288), suggesting that it could be regarded as negative, but at the same time emphasizing the conceptual difficulties of knowing if it is caused by a properly applied treatment or not. Determining what constitutes non-response is also a question that requires a broader theoretical and philosophical discussion about treatment outcomes. Taylor et al. (2012), for instance, described some of the standards that are currently being used for identifying non-response among patients, arguing that these are often based on arbitrary cutoffs, such as a predetermined level of change or a statistical method. There is currently no consensus on how to reliably classify patients as non-responders, with many studies employing some form of diagnostic criteria, while other rely on the change scores that exceed measurement error, i.e., the Reliable Change Index (RCI; Jacobson and Truax, 1991). In a systematic review of CBT for anxiety disorders (including 87 clinical trials and 208 response rates) by Loerinc et al. (2015), the average response rate to treatment was 49.5%. In other words, about half of the patients did not respond or deteriorated. However, they noted significant heterogeneity across studies, suggesting that the response rates differ partly because of how response and non-response are defined. Looking more closely at how this was determined in the specific clinical trials revealed that 31.3% applied the RCI, 70.7% used a clinical cutoff, and 90.9% relied on some change from baseline (of note: several response rates can be used simultaneously in the same clinical trial, hence not adding up to 100%). Similar response rates have also been found in naturalistic settings when applying fixed benchmarks on self-report measures as cutoffs (Gyani et al., 2013; Firth et al., 2015), meaning that it is not uncommon for patients to experience a standstill in their treatment in a regular outpatient health care setting despite receiving the best available care.

During the last two decades, new ways of disseminating evidence-based treatments have been introduced and become an important addition to the regular outpatient health care setting. One of the most widespread formats is Internet-based CBT (ICBT), in which patients complete their treatment via a computer, tablet, or smartphone (Andersson, 2018). Similar to seeing someone face-to-face, reading material and homework assignments are considered essential components and introduced as one module per week. Patients then work on their problem and receive guidance and feedback from a therapist via email, corresponding to what would be discussed during a real-life session (Andersson, 2016). Presently, the efficacy of ICBT has been evaluated in close to 300 randomized controlled trials and several systematic reviews and meta-analyses, demonstrating its benefits for a large number of psychiatric as well as somatic conditions, including in naturalistic settings (Andersson et al., 2019). The results also seem to be maintained over time, with follow-ups at 3 years showing sustained improvements (Andersson et al., 2018). However, like treatments in general, ICBT is not without negative effects. Recent studies have for example shown that 5.8% of patients deteriorate (Rozental et al., 2017), and that a large proportion report adverse and unwanted events (Rozental et al., inpress). Yet, in terms of non-response, few attempts have been made to specifically explore its occurrence and predictors. A notable exception is a study by Boettcher et al. (2014b), investigating negative effects of ICBT for social anxiety disorder. The results showed that the rate of non-responders on the primary outcome measure varied greatly during the treatment period, with 69.9% in mid-treatment, 32.3 at post-treatment, and 29.3% at 4-month follow-up. No attempt at analyzing predictors was however made. In general, the systematic study of non-response has been lacking in relation to ICBT (Andersson et al., 2014), which makes it unclear what factors might be responsible for its incidence and how this information could be used clinically (Hedman et al., 2014).

Considering the fact that a large proportion of all patients do not respond to treatment, the issue of finding those who are at risk of non-response is important. Still, few studies have explicitly explored if non-response can be predicted. Taylor et al. (2012) made an attempt at summarizing the scientific literature, describing three general factors that might prevent a patient from responding. First, poor homework adherence in CBT seems to be predictive of poorer treatment outcome, at least when it is evaluated using a sufficiently reliable and valid measure (Kazantzis et al., 2016). Second, high expressed emotion, i.e., residing in an environment characterized by hostility and emotional over-involvement, is also associated with poorer treatment outcome, but findings are mixed depending on diagnosis. Third, poorer treatment outcome is more likely if the patient displays greater symptom severity at baseline or suffers from a comorbid condition. However, in all of these cases, the focus of the research has been on responders and not explicitly non-responders, meaning that the conclusions are in fact being back-tracked. In addition, information on the standards for determining non-response have not always been clear or lacking completely. This makes it difficult to interpret the results and draw inferences to the study of non-response *per se*, making a more systematic approach to exploring the issue warranted.

Given the scarcity of research on non-response and its predictors the current study thus aims to investigate its occurrence and predictors. Seeing as ICBT is also becoming more and more common in the regular outpatient health care setting, and because it differs somewhat from seeing someone face-to-face (i.e., no or few physical meetings), it could be important to determine how often and why some patients do not seem to respond to this type of treatment. This was done by specifically looking at those patients who do not seem to benefit from ICBT, as determined using different criteria for determining non-response based on the RCI (Jacobson and Truax, 1991), and then applying a set of variables defined a priori in an analysis of possible predictors. In order to complete such a study, a large sample of individual patient data is however needed to ensure adequate statistical power (Oxman et al., 1995). Data from 29 clinical trials is therefore used, aggregated as part of a similar endeavor regarding deterioration rates (Rozental et al., 2017). The data set consists of a total of 2,866 patients, including three categories of diagnoses: anxiety disorders, depression, and other (erectile dysfunction, relationship problems, and gambling disorder). The hypotheses are that non-response rates similar to those reported by Loerinc et al. (2015) will be obtained, i.e., 44.5%. In addition, it is also hypothesized that the findings by Taylor et al. (2012) will be seen in the current study, that is, symptom severity at baseline and module completion, a proxy for homework adherence, will constitute significant predictors of non-response, i.e., increasing the odds of not responding. Lastly, similar to Rozental et al. (2017), not being in a relationship, younger age, and having a lower educational level are also hypothesized to be associated with increased odds of non-response.

## Materials and Methods

### Individual Patient Data Meta-Analysis

To explore the rates and predictors of non-response, individual-level data from many patients are required. The current study consequently conducted an individual patient data meta-analysis, which is a powerful approach of combining the raw scores from each patient variable across studies instead of only relying on group means and standard deviations (Simmonds et al., 2005). This makes it possible to do more sophisticated statistical analyses, particularly when trying to investigate factors that might be predictive of a certain event (Oxman et al., 1995). Similar to a meta-analysis, this can be done either by performing a systematic review or pooling together data from different sites, such as university clinics. The current study used the latter method, aggregating data from those clinical trials that have been conducted by the authors and where the raw scores of patients were possible to obtain. Data from three sites run by the authors were thus screened for eligibility; (1) patients being allocated to a treatment condition involving ICBT, guided or unguided, consisting of treatment interventions that are based on CBT, including applied relaxation and cognitive bias modification (2) meeting the criteria for a psychiatric disorder or V-codes listed in the Diagnostic and Statistical Manual of Mental Disorders, Fourth or Fifth Edition (American Psychiatric Association, 2000, 2013) (3) receiving ICBT that lasted for at least 2 weeks or two modules, and (4) completing a validated primary outcome measure assessing the patients' level of distress, for instance, for social anxiety disorder, this involved the Liebowitz Social Anxiety Scale—Self-Report (LSAS-SR; Liebowitz, 1987). Clinical trials not included in the current study were characterized by treatment conditions other than ICBT, namely, bibliotherapy with telephone support, or treatments that are not theoretically linked to CBT, such as, psychodynamic psychotherapy and interpersonal psychotherapy. A limitation of using this method is of course that it is not possible to assess the risk of bias, such as when implementing a systematic review (Simmonds et al., 2005). However, this allowed the retrieval of a majority of all clinical trials of ICBT that have been executed in Sweden, meaning that it should be representative of how it is being administered on a national level at both university clinics and in a regular outpatient health care setting, i.e., screening patients by diagnostic interviews and distributing validated outcome measures, consistent procedures for guidance by therapists, and similar distribution of treatment content.

Once the clinical trials were selected, the raw scores from each patient were put into the same data matrix and coded for consistency, e.g., sick leave (1 = yes, 0 = no) (i.e., in Sweden, receiving disability checks when being absent from work during a time period of at least 2 weeks up to 1 year due to a medical or psychiatric condition). This includes; name of the clinical trial, treatment condition, and including all available sociodemographic variables, outcome measures (primary and additional), ratings of satisfaction, and credibility, previous use of any type of psychological treatment and previous or ongoing use of psychotropic medication, sick leave, number of completed modules and time spent per week on the treatment interventions. To enable as many comparisons as possible in the statistical analysis, given that clinical trials sometimes used different coding schemes, sociodemographic variables had to be collapsed. For instance, only single/relationship were retained in terms of civil status, while the highest attained educational level was restricted to fewer but more coherent categories. Similarly, diagnoses were re-categorized to balance out their proportions: (1) anxiety disorders, and (2) depression and other (erectile dysfunction, relationship problems, and gambling disorder). Meanwhile, those numbers among the raw scores that were unclear, i.e., when information about a nominal variable was missing, published and unpublished manuscripts were obtained and checked so that the data matrix was coded in accordance with the clinical trials. However, it should be noted that the coding schedules for some of the original datasets were impossible to retrieve, whereby a few cells remained blank. For an overview of the patients' sociodemographic variables and the amount of missing data, see Table 1.

**Table 1:** Sociodemographics variables of patients included in the individual patient data meta-analysis.

**Variable**	**Treatment (*n* = 2,118)**	**Control (*n* = 748)**	**Full sample (*n* = 2,866)**	**Missing data**
Gender: *n* (% female)	1299 (62.1)	501 (67)	1800 (63.4)^a^	27 (0.9)^b^
Age (years): M (*SD*)	38 (12.5)	40.6 (13.2)	38.7 (12.8)	29 (1)
Civil status: *n* (%)				744 (27)
Single	497 (33.5)	171 (28.1)	668 (31.9)	
Relationship	986 (66.5)	438 (71.9)	1424 (68.1)	
Children: *n* (% yes)	554 (53.4)	226 (59)	780 (55)	1,446 (50.5)
Cohabitant: *n* (% yes)	306 (66.5)	48 (69.6)	354 (12.4)	2,337 (81.5)
Highest educational level: *n* (%)				1,099 (38.3)
Elementary school	53 (4.5)	33 (5.7)	86 (4.9)	
High school/college	361 (30.5)	169 (28.9)	530 (30)	
University	757 (64)	380 (65.1)	1137 (64.3)	
Postgraduate	12 (1)	2 (0.3)	14 (0.8)	
Employment: *n* (%)				1,968 (68.7)
Unemployed	74 (10.8)	19 (9)	93 (10.4)	
Student	99 (14.4)	39 (18.6)	138 (15.4)	
Employed	469 (68.2)	138 (65.7)	607 (67.6)	
Other	13 (1.9)	11 (5.2)	24 (2.7)	
Retired	33 (4.8)	3 (1.4)	36 (4)	
Primary diagnosis: *n* (%)				88 (3.1)
Anxiety disorders	1,148 (55.8)	533 (74.1)	1681 (60.5)	
Generalized anxiety disorder	141 (6.8)	138 (19.2)	279 (10)	
Social anxiety disorder	708 (34.4)	257 (35.7)	965 (34.7)	
Anxiety disorder NOS	11 (0.5)	20 (2.8)	31 (1.1)	
Panic disorder (with/without agoraphobia)	86 (4.2)	30 (4.2)	116 (4.2)	
Posttraumatic stress disorder	32 (1.6)	32 (4.5)	64 (2.3)	
Anxiety disorder (with/without depression)	117 (5.7)	56 (7.8)	173 (6.2)	
Specific phobia	53 (2.6)	0 (0)	53 (1.9)	
Depression (with/without dysthymia)	475 (23.1)	69 (9.6)	544 (19.6)	
Other	436 (21.2)	117 (16.3)	553 (19.9)	
Erectile dysfunction	39 (1.9)	39 (5.4)	78 (2.8)	
Relationship problems	80 (3.9)	78 (10.8)	158 (5.7)	
Gambling disorder	317 (15.4)	0 (0)	317 (11.4)	
Sick leave: *n* (% yes)	42 (5.5)	25 (7.6)	67 (6.1)	1,768 (61.7)
Previous psychological treatment: *n* (% yes)	575 (54.1)	214 (56.5)	789 (54.7)	1,424 (49.7)
Previous or ongoing psychotropic medication: *n* (% yes)	366 (31.7)	156 (33.1)	522 (32.1)	1,239 (43.2)
Satisfaction with treatment: M (*SD*)^c^	2.9 (1)	n.a.	2.9 (1)	1,867 (88.2)^e^
Treatment credibility: M (*SD*)^d^	7 (2.4)	n.a.	7 (2.4)	1,535 (72.5)^e^
Modules completed: M (*SD*)^f^	6.5 (1.3)	n.a.	6.5 (1.3)	1,194 (56.4)^e^
Time per week: M (*SD*)^g^	3.6 (3.1)	n.a.	3.6 (3.1)	1,722 (81.3)^e^

*n.a., not applicable; NOS, not otherwise specified*.

a *Valid percent, i.e., percent of available data, excluding missing data*.

b *Percent, i.e., percent of complete dataset, including missing data*.

c *Self-rated 0–5*.

d *Self-rated 0–10*.

e *Based on patients receiving treatment*.

f *Weighted mean and standard deviation*.

g *Number of hours per week*.

### Statistical Analysis

Given the lack of consensus on how to define and determine non-response in treatment (Taylor et al., 2012), the RCI was chosen based on its widespread use and recognition in the scientific literature for assessing reliable change (Jacobson and Truax, 1991). This was calculated by taking the change score on a clinical trial's primary outcome measure for a specific patient and dividing it by the standard error of difference (Speer, 1992), i.e., *SE*_diff_ = *SD*_1_√2√1-*r*, where *SD*_1_ corresponds to the standard deviation of a condition at pre-treatment, and *r* is the reliability estimate (Evans et al., 1998). This calculation also takes into account possible regression to the mean effects and is often referred to as the Edwards and Nunally-method (Speer, 1992). According to Bauer et al. (2004), different ways of calculating the RCI yields similar estimates, but here Speer (1992) was chosen given that it was used in the study of deterioration by Rozental et al. (2017). The RCI was then worked out separately for the primary outcome measure for every clinical trial and using their respective test-retest reliability rather than internal consistency (see Table 2), in line with the recommendations by Edwards et al. (1978). Essentially, the RCI sets the boundaries for which a change score can be deemed reliable, meaning that it would be unlikely (*p* = 0.05), without a true change actually occurring. For example, considering the first clinical trial in the current study, IMÅ, a change score of ±10.13 is considered reliable on the Beck Anxiety Inventory (Beck et al., 1988). A change score that does not exceed ± 10.13 would then be deemed as non-response. Hence, using the RCI in this way, the change scores for the primary outcome measure in each clinical trial and for each patient was used to classify non-responders, which were dummy coded into a nominal variable (1 = yes, 0 = no). However, it should be noted that a RCI is usually calculated on the basis of a standard deviation unit of change equal to *z* = 1.96. Wise (2004) argued that this is a relatively conservative estimate, at least for investigating improvement and deterioration, proposing reliable change indexes that represents different confidence levels, i.e., *z* = 1.28 for a moderate change and *z* = 0.84 for a minor change. Although affecting the probability of rejecting the null hypothesis, *p* = 0.10 and 0.20, this could be useful for detecting less frequently occurring events, such as deterioration, or to make the boundaries of the RCI narrower, as in non-response (i.e., a smaller change score would be required to be classified as a responder, consequently affecting the non-response rate). Again, using the clinical trial IMÅ as an example, a change score of ± 6.62 is regarded as a reliable change for *z* = 1.28, and 4.94 for *z* = 0.84. In the current study, the non-response rates for each clinical trial and the total estimates are presented for each of the reliable change indexes in order to facilitate a comparison, while only *z* = 1.96 is applied for analyzing possible predictors as it should increase power. All of the non-response rates are based on data for patients receiving treatment and not some form of control condition.

**Table 2:** Test-retest reliabilities used for calculating the RCI.

**Primary outcome**	**Test-retest reliability**	**Time period**	**Population**	**References**
Beck Anxiety Inventory	*r* = 0.81	2 weeks	Normal	Saemundsson et al., 2011
Liebowitz Social Anxiety Scale—Self-Report	*r* = 0.93	8 weeks	Normal	Heeren et al., 2012
Panic Disorder Severity Scale—Self-Report	ρ = 0.94	2 days	Patient	Lee et al., 2009
Patient Health Questionnaire−9 Items	*r* = 0.94	2 weeks	Patient	Zuithoff et al., 2010
International Index of Erectile Functioning−5 Items^a^	*r* = 0.84	4 weeks	Patient	Rosen et al., 1997
Beck Depression Inventory	*r* = 0.77	^b^	Normal	Beck and Steer, 1996
Impact of Event Scale—Revised	*r* = 0.89-0.94^c^ *M* = 0.92	6 months	Patient	Sundin and Horowitz, 2002
Generalized Anxiety Disorder−7 Items	ICC = 0.83	1 week	Patient	Spitzer et al., 2006
Penn State Worry Questionnaire	*r* = 0.84	3 weeks	Normal	Pallesen et al., 2006
Body Sensations Questionnaire	*r* = 0.89	3 months	Patient	Arrindell, 1993
Dyadic Adjustment Scale^a^	*r* = 0.87	2 weeks	Patient	Carey et al., 1993
Snake Anxiety Questionnaire	*r* = 0.78	1 month	Normal	Klorman et al., 1974
Montgomery-Åsberg Depression Rating Scale—Self-Report	ICC = 0.78	1 week	Patient	Fantino and Moore, 2009
Spider Phobia Questionnaire	*r* = 0.94	3 weeks	Normal	Muris and Merckelbach, 1996
The NORC Diagnostic Screen for Gambling Problems	*r* = 0.98–0.99^d^ *M* = 0.98	1 week	Patient	Gerstein et al., 1999

*RCI, reliable change index; NORC, a National Organization for Research at the University of Chicago*.

a *Reversed scales, higher scores indicate less problems*.

b *Information regarding the time period was unavailable*.

c *Separate estimates for the two subscales*.

d *Lifetime test statistic and past year test statistic*.

To investigate possible predictors, binomial logistic regression was applied with the dichotomized coding of non-response (1 = yes, 0 = no) used as the dependent variable. All predictors were entered into the model in one single block as independent variables, as no prior evidence exist with regard to building the model. However, in terms of choosing what variables to enter, theoretical assumptions or empirical findings were used as guidance to avoid the risk of finding spurious associations and restrict the type-I-error rate (Stewart and Tierney, 2002). Hence, the same variables used for investigating the predictors of deterioration were implemented (Rozental et al., 2017): (1) symptom severity at baseline, (2) civil status, (3) age, (4) sick leave, (5) previous psychological treatment, (6) previous or ongoing psychotropic medication, (7) educational level, and (8) diagnosis. Two *post-hoc* and explorative variables were also entered: (9) gender, and (10) module completion. Both symptom severity at baseline and module completion, a proxy for homework adherence, have been put forward as predictors for non-response (Taylor et al., 2012). Meanwhile, albeit not specifically linked to non-response, male gender, lower age, and lower educational level have previously been shown to predict dropout in ICBT (Christensen et al., 2009; Waller and Gilbody, 2009; Karyotaki et al., 2015).

Predictors with a *p* < 0.05 were regarded as significant and presented as Odds Ratios (OR) with their respective 95% Confidence Intervals (CI), reflecting an increase or decrease in odds of non-response in relation to a reference category. For instance, for dichotomous predictors such as sick leave, the OR reflects the adjustment in odds of non-response when the patient goes from not being on sick leave (no) to being on sick leave (yes). For the three predictors that were on continuous scales, that is, symptom severity at baseline, age, and module completion, the OR represents an increase of one standard deviation above their respective mean, i.e., these variables were standardized and centered within each clinical trial. All statistical analyses were performed using jamovi version 0.9.2.9 (Jamovi project, 2018), and on a complete case basis given that it is unclear how missing data should be treated when investigating non-response.

### Ethical Considerations

The data in the current study were aggregated from several clinical trials, all with written informed consent, and all having received ethical approval from the Regional Ethical Review Board at their respective study location (please refer to the original articles for more information). The data included only the raw scores from various patient variables and no sensitive or qualitative information. Moreover, all patients were given an automatically assigned identification code in each clinical trial, e.g., abcd1234, making it impossible to identify a particular individual. In terms of the ethical issue related to the assessment of non-response in ICBT, the current study used only the raw scores from already completed clinical trials, making it impossible to, in hindsight, detect and help patients that may not have benefitted from treatment. However, because the aim of the current study is to explore the occurrence and possible predictors of non-response, future clinical trials may be better able to monitor and assist those patients who are not responding.

## Results

### Study Characteristics

Data from 29 clinical trials were reviewed according to predefined inclusion and exclusion criteria and deemed eligible for the current study. Raw scores from all patients were then entered into the data matrix. In total, 2,118 (73.9%) had received treatment (ICBT). The following diagnoses were included (clinical trials, *k*): social anxiety disorder (*k* = 9), depression (with/without dysthymia; *k* = 5), generalized anxiety disorder (*k* = 3), anxiety disorder (with/without depression; *k* = 3), mixed anxiety disorders (e.g., panic disorder as well as social anxiety disorder; *k* = 2), specific phobia (*k* = 2), post-traumatic stress disorder (*k* = 1), panic disorder (with/without agoraphobia; *k* = 1), gambling disorder (*k* = 1), erectile dysfunction (*k* = 1), and relationship problems (*k* = 1). In terms of recruitment, self-referrals from the general population were most common, 27 clinical trials, but one was conducted in primary care, and another at a university clinic. With regard to screening interviews, the Structured Clinical Interview for DSM-IV-Axis I Disorders (First et al., 1997), was mostly applied, followed by four clinical trials that implemented either the MINI-International Neuropsychiatric Interview (Sheehan et al., 1998), or a diagnosis-specific instrument, e.g., Clinician-Administered PTSD Scale (Blake et al., 1995). The length of treatment ranged from four to 10 modules (*M* = 8.28; *SD* = 1.36), 4–12 weeks (*M* = 8.45; *SD* = 1.66), and two to 10 sessions (*M* = 5.40; *SD* = 3.58), with specific phobia being shortest, while various anxiety disorders and relationship problems were the longest. The total amount of missing data for the primary outcome measures at post-treatment was 12.9%. For a complete overview of the clinical trials, please refer to Table 3.

**Table 3:** Characteristics and non-response rates for the clinical trials included in the individual patient data meta-analysis.

**Study**	**Recruitment**	**Screening interview**	**Primary diagnosis**	**Treatment (*n*)**	**Control (*n*)**	**Modules/weeks or sessions**	**Primary outcome**	**Additional outcomes**	**Non-response 1.96 *n* (%)**	**Non-response 1.28 *n* (%)**	**Non-response 0.84 *n* (%)**
IMÅ (Boettcher et al., 2014a)	General population	SCID-I	Panic disorder, social anxiety disorder, generalized anxiety disorder, anxiety disorder NOS	Unguided mindfulness with FAQ (42)	Wait-list with discussion forum (46)	8 modules/8 weeks	BAI	BDI, QOLI, ISI	12 (28.6%)	8 (19.0%)	5 (11.9%)
ACT Smart (Lindner et al., 2013)	General population	SCID-I	Panic disorder, social anxiety disorder	Unguided ACT (48), guided ACT (48)	Wait-list (47)	8 modules/10 weeks	LSAS-SR, PDSS-SR^a^	PHQ-9, GAD-7, QOLI	36 (37.5%)	25 (26.0%)	17 (17.7%)
ACTUA (Nyström et al., 2017)	General population	SCID-I	Depression	Guided physical activity (164), guided behavioral activation (158)^b^	n.a.	8 modules/12 weeks	PHQ-9	GAD-7, QOLI^c^	35 (10.9%)	15 (4.7%)	15 (4.7%)
ADAM (Andersson et al., 2011)	General population	Semi-structured interview, IIEF-5	Erectile dysfunction	Guided CBT (39)	Wait-list with discussion forum (39)	7 modules/7 weeks	IIEF-5	IIEF, RAS, BDI, BAI	29 (74.4%)	22 (56.4%)	18 (46.2%)
Challenger (Boettcher et al., 2018)	General population	MINI	Social anxiety disorder	Unguided CBT with smartphone application (68), unguided bibliotherapy (70)	Wait-list (69)	9 modules/6 weeks	LSAS-SR	GAD-7, PHQ-9, QOLI, BBQ, Mini-SPIN	46 (33.3%)	26 (18.8%)	19 (13.8%)
Stella (Andersson et al., 2013a)	General population	SCID-I & II	Depression	Guided CBT (33), group therapy (36), guided CBT as preferred choice (16)	n.a.	8 modules/8 weeks, 8 sessions	BDI	MADRS-S, BAI, HAM-D, QOLI,	47 (55.3%)	34 (40.0%)	18 (21.2%)
Depressionshjälpen (Carlbring et al., 2013)	General population	SCID-I	Depression	Guided CBT (40)	Wait-list (40)	7 modules/8 weeks	BDI	MADRS-S, BAI, QOLI, WAI	13 (32.5%)	8 (20.0%)	7 (17.5%)
Tellus (Ivarsson et al., 2014)	General population	CAPS	Posttraumatic stress disorder	Guided CBT (31)	Wait-list with support (31)^d^	8 modules/8 weeks	IES-R	PDS, BDI, BAI, QOLI	6 (19.4%)	3 (9.7%)	3 (9.7%)
Klara (Vernmark et al., 2010)	General population	SCID-I	Depression	Guided CBT (29), guided CBT via email (30)	Wait-list (29)	7 modules/8 weeks	BDI	MADRS-S, BAI, QOLI	8 (13.6%)	7 (11.7%)	3 (5.1%)
Oroshjälpen (Dahlin et al., 2016)	General population	SCID-I	Generalized anxiety disorder	Guided ACT (52)	Wait-list (51)	7 modules/9 weeks	GAD-7	PSWQ, GAD-Q-IV, BAI, MADRS-S, PHQ-9, QOLI	14 (26.9%)	5 (9.6%)	3 (5.8%)
Nova 1 (Carlbring et al., 2011)	General population	SCID-I	Anxiety disorder with/without comorbid depression	Guided CBT (53)	n.a.	10 modules/10 weeks^e^	BAI	MADRS-S, CORE-OM, QOLI	30 (56.6%)	21 (39.6%)	13 (24.5%)
Nova 2 (Nordgren et al., 2014)	Primary care	SCID-I	Anxiety disorder with/without comorbid depression	Guided CBT (51)	Wait-list (50)	10 modules/10 weeks^f^	BAI	MADRS-S, CORE-OM, QOLI	30 (58.8%)	20 (39.2%)	10 (19.6%)
Origo 1 (Almlöv et al., 2011)	General population	SCID-I	Generalized anxiety disorder	Guided CBT (44)	Wait-list (45)	8 modules/8 weeks	PSWQ	GAD-Q-IV, STAI, BAI, BDI, MADRS-S, QOLI	15 (34.1%)	10 (22.7%)	6 (13.6%)
Origo 2 (Andersson et al., 2012)	General population	SCID-I	Generalized anxiety disorder	Guided CBT (27)^g^	Wait-list (27)	8 modules/8 weeks	PSWQ	GADQ-IV, MADRS-S, BDI, BAI, STAI, QOLI	10 (37.0%)	10 (37.0%)	10 (37.0%)
Panik 2 (Carlbring et al., 2005)	General population	SCID-I, CIDI	Panic disorder	Guided CBT (25), face-to-face CBT (24)	n.a.	10 modules/10 weeks, 10 sessions	BSQ	ACQ, MI, BAI, BDI, QOLI	12 (24.5%)	5 (10.2%)	1 (2.0%)
Pia (unpublished)	General population	SCID-I	Relationship problems	Guided CBT (80)	Wait-list with discussion forum (78)	10 modules/10 weeks	DAS	MSI, BDI, BAI, QOLI	40 (50.0%)	25 (31.3%)	17 (21.3%)
Progredi, (Ström et al., 2013)	General population	SCID-I	Depression	Guided CBT with physical activity (24)	Wait-list (24)	9 modules/9 weeks	MADRS-S	BDI, BAI, QOLI^c^	7 (29.2%)	3 (12.5%)	1 (4.2%)
Fobal orm (Andersson et al., 2013b)	General population	SCID-I	Specific phobia	Guided CBT (13), face-to-face CBT (13)	n.a.	4 modules/4 weeks, 2 sessions^h^	SNAQ	ADIS, FSS, BAI, BDI	2 (7.6%)	1 (3.9%)	1 (3.9%)
Sofie 13 (Boettcher et al., 2013)	General population	SCID-I	Social anxiety disorder	Guided CBT with CBM (61), Guided CBT without CBM (65),	n.a.	9 modules/9 weeks^i^	LSAS-SR	SIAS, SPS, MADRS-S, QOLI	26 (20.6%)	12 (9.5%)	4 (3.2%)
Sofie 9 (Kuckertz et al., 2014)	General population	SCID-I & II	Social anxiety disorder	Guided CBT (40)	Attention bias modification (39)	9 modules/9 weeks	LSAS-SR	SIAS, SPS, SPSQ, BAI, MADRS-S, QOLI	9 (22.5%)	7 (17.5%)	3 (7.5%)
Sofie 12 (Dagöö et al., 2014)	General population	SCID-I, MINI	Social anxiety disorder	Guided CBT with smartphone application (24)^j^	n.a.	9 modules/9 weeks	LSAS-SR	^k^	6 (25.0%)	2 (8.3%)	1 (4.2%)
Sofie 1 (Andersson et al., 2006)	General population	SCID-I	Social anxiety disorder	Guided CBT (32)	Wait-list (32)	9 modules/9 weeks	LSAS-SR	SIAS, SPS, SPSQ, BAI, MADRS-S, QOLI	7 (21.9%)	4 (12.5%)	2 (6.3%)
Sofie 2 (Carlbring et al., 2007)	General population	SCID-I	Social anxiety disorder	Guided CBT (29)	Wait-list (28)	9 modules/9 weeks	LSAS-SR	SIAS, SPS, SPSQ, BAI, MADRS-S, QOLI,	10 (34.5%)	6 (20.7%)	4 (13.8%)
Sofie 3 (Tillfors et al., 2008)	Students	SCID-I	Social anxiety disorder	Guided CBT (19), guided CBT with group sessions (18)	n.a.	9 modules/9 weeks, 5 sessions	LSAS-SR	SIAS, SPS, SPSQ, BAI, MADR-S, QOLI	16 (43.2%)	12 (32.4%)	11 (29.7%)
Sofie 4 (Furmark et al., 2009)	General population	SCID-I & II	Social anxiety disorder	Guided CBT with discussion forum (40), unguided bibliotherapy (40)	Wait-list (40)	9 modules/9 weeks	LSAS-SR	SIAS, SPS, SPSQ, BAI, MADR-S, QOLI	34 (42.5%)	20 (25.0%)	12 (15.0%)
Sofie 5 (Furmark et al., 2009)	General population	SCID I	Social anxiety disorder	Guided AR (29), guided CBT with discussion forum (29), unguided bibliotherapy (29), unguided bibliotherapy with discussion forum (28)	n.a.	9 modules/9 weeks	LSAS-SR	SIAS, SPS, SPSQ, BAI, MADR-S, QOLI	37 (32.2%)	21 (18.3%)	12 (10.4%)
Elsa (in preparation)	General population	SCID-I	Anxiety disorder with/without comorbid depression	Guided CBT (33)	Wait-list with support (33)	8 modules/8 weeks^l^	BAI	MADRS-S, CORE-OM, PHQ-9, GAD-7, QOLI	16 (48.5%)	12 (36.4%)	12 (36.4%)
Fobal spindel (Andersson et al., 2009)	General population	SCID-I	Specific phobia	Guided CBT (13), face-to-face CBT (14)	n.a.	5 modules/4 weeks, 2 sessions^h^	SPQ	ADIS, FSS, BAI, BDI	3 (11.1%)	1 (3.7%)	0 (0.0%)
Gamble (Carlbring et al., 2012)	General population	n.a.	Gambling disorder	Guided CBT with discussion forum (317)	n.a.	8 modules/8 weeks	NODS	HADS, QOLI	11 (3.5%)	11 (3.5%)	11 (3.5%)
Total non-response rates									567 (26.8%)	356 (16.8%)	239 (11.3%)

*SCID-I, Structural Clinical Interview for DSM-IV Axis I Disorders; NOS, Not Otherwise Specified; FAQ, Frequently Asked Questions; BAI, Beck Anxiety Inventory; BDI, Beck Depression Inventory; QOLI, Quality of Life Inventory; ISI, Insomnia Severity Index; n.a., not applicable; ACT, Acceptance and Commitment Therapy; LSAS-SR, Liebowitz Social Anxiety Scale–Self-Report; PDSS-SR, Panic Disorder Severity Scale–Self-Report; PHQ-9, Patient Health Questionnaire−9 Items; GAD-7, Generalized Anxiety Disorder−7 Items; BA, Behavioral Activation; MADRS-S, Montgomery-Åsberg Depression Rating Scale–Self-Report; IIEF-5, International Index of Erectile Functioning−5 Items; CBT, Cognitive Behavior Therapy; IIEF, International Index of Erectile Functioning; RAS, Relationship Assessment Scale; MINI, The MINI-International Neuropsychiatric Interview; BBQ, Brunnsviken Brief Quality of Life Inventory; Mini-SPIN, Mini-Social Phobia Inventory; HAM-D, Hamilton Rating Scale for Depression; WAI, Working Alliance Inventory; CAPS, Clinician-administered PTSD Scale for DSM-IV; IES-R, Impact of Event Scale–Revised; PDS, Posttraumatic Diagnostic Scale; PSWQ, Penn State Worry Questionnaire; GAD-Q-IV, Generalized Anxiety Disorder Questionnaire; CORE-OM, Clinical Outcome in Routine Evaluation–Outcome Measure; STAI, State-Trait Anxiety Inventory; CIDI, Composite International Diagnostic Interview; ACQ, Agoraphobic Cognitions Questionnaire; BSQ, Body Sensations Questionnaire; MI, Mobility Inventory; DAS, Dyadic Adjustment Scale; MSI, Marital Status Inventory; SNAQ, Snake Anxiety Questionnaire; ADIS, Anxiety Disorders Interview Schedule; FSS, Fear Survey Schedule; CBM, Cognitive Bias Modification; SIAS, Social Interaction Anxiety Scale; SPS, Social Phobia Scale; SPSQ, Social Phobia Screening Questionnaire; AR, Applied Relaxation; SPQ, Spider Phobia Questionnaire; NODS, The NORC Diagnostic Screen for Gambling Problems*.

a *Separate analyses of deterioration were conducted for the two primary outcome measures depending on the diagnosis of the patient*.

b *Four treatment conditions were included in the study, with/without treatment rationale, respectively, but are pooled in the current analysis*.

c *An additional outcome measure, International Physical Activity Questionnaire, IPAQ, was used in the study, but is not included in the current analysis*.

d *Passive control with the possibility to contact the research team if needed*.

e *Patients were able to choose ten out of 16 modules to be completed during ten weeks*.

f *Patients were able to choose ten out of 19 modules to be completed during ten weeks*.

g *An additional treatment group, guided psychodynamic therapy, was also used in the study, but is not included in the current analysis*.

h *One brief orientation session and one session of three-hour prolonged exposure*.

i *In addition to two weeks of CBM*.

j *An additional treatment group, interpersonal psychotherapy, was also used in the study, but is not included in the current analysis*.

k *Additional outcome measures were included in the original study but lost in the raw data file*.

l *Patients were able to complete up to eight modules selected by the therapist*.

### Non-response Rates

Of the 2,118 patients, 567 (26.8%) were classified as non-responders when using a RCI of *z* = 1.96. In comparison, the numbers were a bit lower, 356 (16.8%) for *z* = 1.28, and a mere 239 (11.3%) for *z* = 0.84, indicating that the non-response rates vary depending on what reliable change indexes are being employed, each step being statistically significant, χ^2^_(2)_ = 64.89, *p* < 0.05, and χ^2^_(2)_ = 27.57, *p* < 0.05. The lowest rates of non-response (*z* = 1.96) can be found in clinical trials for gambling disorder (3.5%), specific phobia for snakes (7.6%), and depression (10.9%). Meanwhile, the highest rates were obtained in clinical trials on erectile dysfunction (74.4%), and anxiety disorders (with/without comorbid depression; 58.8 and 56.6%, respectively). See Table 3 for an outline of the non-response rates in each clinical trial, sorted according the respective reliable change indexes.

### Predictors of Non-response

A binomial logistic regression was performed with the predefined variables entered as predictors for non-response. The results can be seen in Table 4, together with their respective OR and 95% CI. Overall, the output seems to suggest that patients receiving treatment had increased odds of non-response if they had higher symptom severity on the primary outcome measure at baseline. Similarly, there were increased odds for not responding in treatment when having an anxiety disorder as compared to depression and other (erectile dysfunction, relationship problems, and gambling disorder), and if the patient was of male gender. None of the other variables were predictive of non-response.

**Table 4:** Significance level, odds ratios, and 95% Confidence Intervals (CI) for predictors of non-response.

**Predictor (reference)**	***p***	**OR**	**Lower CI**	**Upper CI**
Symptom severity at baseline (lower severity)	<0.001	2.04	1.53	2.72
Diagnosis, anxiety disorders (depression and other)	<0.001	5.75	2.92	11.32
Module completion (fewer)	0.12	1.09	0.98	1.22
Civil status (single)	0.13	0.66	0.39	1.13
Gender (female)	0.03	1.80	1.05	3.10
Previous psychological treatment (no)	0.99	1.01	0.57	1.77
Previous or ongoing psychotropic medication (no)	0.08	1.72	0.94	3.14
Sick leave (no)	0.51	0.54	0.08	3.45
Educational level (below university level)	0.49	1.20	0.71	2.04
Age (lower age)	0.73	1.00	0.98	1.02

*p, p-value; OR, odds ratio; CI, 95% confidence interval*.

## Discussion

The current study examined the occurrence of non-response in clinical trials of ICBT for three categories of problems including anxiety disorders, depression, and other (erectile dysfunction, relationship problems, and gambling disorder). In total, 2,118 patients in 29 clinical trials received treatment and were analyzed, indicating that 567 (26.8%) were classified as non-responders when using a RCI of *z* = 1.96, but fewer when implementing a narrower criterion, 356 (16.8%) for 1.28, and 239 (11.3%) for 0.84. This goes against the initial hypothesis of finding a similar estimate as the systematic review of CBT for anxiety disorders by Loerinc et al. (2015), which found an average response rate of 44.5%, indicating that non-response could be less frequent in ICBT. However, concluding that non-response is more common in CBT is highly speculative given that such numbers may not be possible to back-track, i.e., the opposite of response also includes patients who deteriorate. Thus, it would be more correct to compare it to attempts at determining non-response more directly. For example, Gyani et al. (2013) demonstrated that 29.0% did not respond among 19,395 patients receiving treatment within Improving Access to Psychological Therapies (IAPT) in the United Kingdom, with a majority undergoing CBT. Similarly, Firth et al. (2015) analyzed 6,111 patients from IAPT using the same method, demonstrating that 32–36% were classified as non-responders. Hence, at least according to these estimates, the rate obtained in the current study on ICBT closely resemble those for treatments delivered face-to-face, at least when using a RCI of *z* = 1.96. However, in these two cases a composite measure of non-response was in fact used, incorporating both the Patient Health Questionnaire-9 Items (PHQ-9; Löwe et al., 2004) and the Generalized Anxiety Disorder Assessment-7 Items (GAD-7; Spitzer et al., 2006). In addition, they also applied a predetermined cutoff for distinguishing responders from non-responders, which is quite different from using the RCI as it only sets one boundary, i.e., determining non-response based on having a treatment outcome *above* a certain threshold as compared to a change score *within* a particular range. In comparison, Hansen et al. (2002) used the RCI for the Outcome Questionnaire-45 (OQ-45; Lambert et al., 1996), i.e., “*no change*, meaning a patient's OQ-45 score had not changed reliably in any direction over the course of therapy” (p. 337), having a non-response rate of 56.8%. Meanwhile, Mechler and Holmqvist (2016) used the RCI in relation to the Clinical Outcomes in Routine Evaluation—Outcome Measure (Evans et al., 2002), with non-response rates being 61.2–66.6% (the range in the latter depending on whether patients were in primary care or a psychiatric outpatient unit). The number of patients not responding to treatment thus seems to vary greatly depending on how this is being classified, making it difficult to draw any definite conclusions on what estimates may be more accurate. This is especially true when different studies use different categories of treatment outcome, such as when improvement is also divided into improved and recovered, thereby obfuscating the results and making direct comparisons more complicated. In addition, it is important to keep in mind what population was explored. Patients in naturalistic settings may differ from those in clinical trials, where inclusion and exclusion criteria may prevent the most severe patients from being included, hence the much higher rates. The numbers from the current study should thus be interpreted cautiously and perhaps only be compared to patients who receive treatment in a tightly controlled research setting where the internal validity is increased and the samples highly selected.

As for ICBT more specifically, comparing non-response rates is difficult. Systematic reviews have not yet explicitly investigated the issue and clinical trials do not generally determine non-responders as a separate categorical outcome. However, a few exceptions exist. Boettcher et al. (2014b) found that 32.2% of the patients receiving CBT via the Internet for social anxiety disorder did not respond when analyzing the primary outcome measure and using the RCI with an intention-to-treat principle. Likewise, Probst et al. (2018) showed that in a treatment for tinnitus distress, 20.4% could be identified as non-responders (27.2% if using an intent-to-treat analysis where missing data was classified as non-response), although, in this case, a predetermined cutoff was utilized. Based only on these examples, findings from the current study seem to be similar, but it would be useful if future clinical trials reported non-response rates more regularly to facilitate systematic reviews.

The current study also looked at how the application of different reliable change indexes affected the non-response rate, demonstrating a range of 15.5% between the widest and narrowest criterion. This approach was based on the recommendations by Wise (2004), contending that it can be useful to assess treatment outcome using different confidence levels: “…would be of considerable help in more accurately identifying and studying those who are not unequivocal treatment successes but who are nonetheless improving and on their way to a positive outcome as well as those who are not responding to treatment.” (p. 56). However, this approach was primarily proposed for improvement and deterioration, while it is less clear if it should be applied to non-response. According to Loerinc et al. (2015), the RCI also seems to be one of the less frequently used classifications of non-response, with only one-third of the clinical trials using it in their systematic review. The results presented here are therefore tentative and need to be replicated, but they do warrant some caution as to how non-response rates are interpreted in the scientific literature (Taylor et al., 2012). Moreover, different reliable change indexes result in different rates of non-response, but what standard deviation unit of change might be most accurate depends on theory and reliability, i.e., is almost two standard deviations too broad a measure of non-response? Looking closer at one of the clinical trials included in the current study, Sofie 1, a change score within ±15.79 on the LSAS-SR (Liebowitz, 1987) classifies a patient as a non-responder when using a RCI of *z* = 1.96, but only 6.77 points for 0.84, thereby decreasing the non-response rate from 21.9 to 6.3%. More research is needed to explore what level is clinically meaningful, that is, when a statistically determined non-response is in fact seen as something negative by the patient. This could, for instance, include interviewing those who do not respond according to the RCI regarding their experiences of treatment, similar to the study by McElvaney and Timulak (2013) who addressed the issue of good and poor outcomes using a qualitative approach.

Lastly, the current study examined possible predictors of non-response in ICBT by entering a set of variables determined a priori into a binomial logistic regression. The results from this analysis suggest that patients with higher symptom severity on the primary outcome measure at baseline, having an anxiety disorder, and being of male gender might have higher odds of not responding in treatment. The fact that greater symptoms may be a predictor is not particularly surprising given that it implies more distress and potential comorbidity, similar to what was proposed by Taylor et al. (2012), which is also in line with the initial hypothesis. Higher symptom severity could also be a sign to extend the treatment period to achieve adequate treatment dosage for those patients who do not improve as expected (Stulz et al., 2013), which is seldom possible in clinical trials. As for anxiety disorders possibly being predictive of non-response, the evidence is less clear. No direct comparisons between diagnoses have previously been made for any treatment, making it difficult to evaluate if and why this would increase the odds for not responding. One idea is that non-response occurs more often among patients with anxiety disorders in ICBT because it is more difficult for a therapist to notice and adjust the treatment without a face-to-face contact (Bengtsson et al., 2015), such as when exposure exercises need to be tweaked to target the correct stimulus or more help is required to increase motivation. Meanwhile, treating depression via the Internet might be more straightforward for the patient and therefore less probable to result in non-response. However, these findings are among the first of its kind and need to be replicated before any definitive conclusions can be drawn. It should also be noted that the third category of diagnoses, other, only consisted of three randomized clinical trials. Still, both erectile dysfunction and relationship problems had among the highest rates of non-response in the current study (74.4 and 50%), which is similar to what was found for deterioration (Rozental et al., 2017), but gambling disorder did on the other not display the same pattern (3.5%). Further research is thus warranted to see if certain diagnoses are more likely to predict non-response in ICBT. Finally, none of the other hypotheses were confirmed, i.e., module completion, not being in a relationship, younger age, and having a lower educational level were not associated with higher odds of non-response. However, being of male gender could constitute a potential predictor, which is in line with the results by Karyotaki et al. (2015) indicating that men tend to drop out from ICBT. Here, a possible difference in coping strategies was proposed as an explanation, where women may put in more effort in trying to overcome their distress, thereby exhibiting a better compliance in treatment. If this somehow also explains the difference in non-response between the genders in ICBT remains to be seen. Yet it could be that male patients have different expectations of what the treatment entails, resulting in poorer response and dropout when these are not met, something that would be interesting to explore in the future via interviews.

### Limitations

The current study is relatively unique in that it has explicitly investigated non-response in treatment and the first using individual patient data meta-analysis. This is considered a gold standard for examining effects above those found by using group means and standard deviations, particularly in relation to discovering potential predictors (Simmonds et al., 2005). However, there are several limitations that need to be considered when interpreting the results. First, few similar examples exist in the scientific literature, making it somewhat difficult to interpret both the rates of non-response and its predictors, especially since there exists no consensus on how to define and classify patients who do not respond. The findings should therefore be interpreted cautiously and warrant replications, although they might help inform researchers of what estimates to expect and variables to explore (Clarke, 2005). Here, a particular caution should be made with regard to the OR's that have been provided, as they may be difficult to interpret and use clinically. In essence, they represent a probability of an event, similar to how odds are used in betting, but cannot be directly translated into a risk of something occurring in the future (Davies et al., 1998). Also, using binomial logistic regression in investigating predictors poses several challenges, such as how to deal with continuous scales, multicollinearity, and the assumptions regarding the relationship between the independent and dependent variables. Second, the current study consists of data from 29 clinical trials with 2,118 patients receiving treatment (2,866 in total), but the aggregation was not based on a systematic review, which could introduce different biases (Stewart and Tierney, 2002), e.g., availability bias and reviewer bias. However, the authors went to great length to ensure that all available data was used and set up predefined inclusion and exclusion criteria as a way of tackling these issues (Rozental et al., 2017). Nevertheless, this means that the results should be explored in additional context, particularly since the clinical trials included in the current study do not have to be representative of how ICBT is conducted in other settings. Third, the patients receiving treatment can be seen as characteristic of most examples of ICBT (Titov et al., 2010), but are nonetheless more often women, in their late thirties, and having a higher education level. However, compared to treatment face-to-face, this is not particularly uncommon either (Vessey and Howard, 1993), probably reflecting a greater tendency to seek help for mental health problems among this group. Still, it does limit the generalizability of the results, particularly in terms of finding predictors of non-response. Future research should thus include patients with a more heterogeneous sociodemographic background and who have not only been self-recruited to clinical trials. This problem is also relevant regarding the diagnoses that were analyzed. Albeit including a broad spectrum of conditions, some were over-represented, e.g., social anxiety disorder, while others were less represented or even lacking completely, e.g., post-traumatic stress disorder and obsessive-compulsive disorder. Depression and other (erectile dysfunction, relationship problems, and gambling disorder) were also re-categorized to balance out their proportions, which risks losing valuable information as to where the difference lies. Thus, it is probably premature to suggest that anxiety disorders constitute a predictor for non-response before a more comprehensive investigation has been made. Fourth, the implementation of the RCI as a way of determining non-response is not without criticism and should be seen as a major limitation. It is presently unclear whether it is the best way to identify those patients who do not respond in treatment, even if there exist a statistical rationale for its use. Furthermore, although the current study followed the recommendations by Edwards et al. (1978) on establishing valid test-retest reliabilities from the literature to calculate the RCI, most estimates relied on relatively short time periods, e.g., 2–4 weeks. This might be more relevant for assessing deterioration or improvement, but not for non-response which may need to take into account longer time frames to determine the natural fluctuation of a diagnosis. It could also be argued that the application of a cutoff or diagnostic criterion is more clinically relevant. However, those thresholds might be more useful in relation to response than non-response, i.e., defining when a patient goes from a clinical to a non-clinical population (Jacobson and Truax, 1991). Predefined numbers, such as being above a certain score, also tend to be arbitrary (Taylor et al., 2012). Still, the use of the RCI to assess non-response needs to be validated by other means. This can for instance be performed by checking if a non-responding patient still fulfills diagnostic criteria or a clinician-rating remains unchanged, e.g., the Clinical Global Impressions Scale (Busner and Targum, 2007). Non-response should also be explored in a direct comparison to deterioration and improvement in future systematic reviews. This is due to the fact that non-response is a quite heterogenous category that could include both those patients who fare worse and achieve some positive results, even though they are, statistically speaking, seen as non-responders. Lastly, the idea of non-response representing a negative effect is not clear and warrants further debate. Both Dimidjian and Hollon (2010) and Linden (2013) argued that it might prolong an ongoing condition and prevent the patient from seeking a more helpful treatment, but that it is also important to consider the normal fluctuations of many diagnoses. In most cases, lack of improvement would probably be regarded as a failure, at least by a clinician. On the other hand, with regard to more serious conditions, lack of improvement may not necessarily be equated with something detrimental for the patient, but rather a perfectly reasonable result, i.e., remaining at a certain level of functioning in chronic pain. Also, as discussed by Linden (2013), non-response does not have to be linked to treatment, but rather other circumstances that occur simultaneously. In sum, regarding non-response as a negative effect clearly needs a discussion that considers not only the approach to classifying patients as non-responders, but also a broader theoretical and philosophical perspective of treatment outcome.

## Conclusions

Among 2,118 patients in 29 clinical trials receiving treatment, 567 (26.8%) were identified as non-responders in ICBT when applying a RCI of *z* = 1.96. This is somewhat in line with other investigations in the scientific literature, although the lack of consensus on how to define non-response make it difficult to compare the results. Meanwhile, possible predictors were explored using variables set a priori, indicating that patients with higher symptom severity on the primary outcome measure at baseline, having an anxiety disorder, and being of male gender could potentially have higher odds of not responding in ICBT. However, additional research is required to replicate the findings and to determine how to best classify non-response in treatment.

## Data Availability

The data that support the findings of this study are available from the corresponding author, [PC], upon reasonable request.

## Author Contributions

All the authors contributed in the process of completing the current study and writing the final manuscript. AR conducted the aggregation of raw scores into a single data matrix and completed the statistical analysis with input from GA and PC. AR drafted the first version of the text, while GA and PC provided feedback and reviewed and revised it.

### Conflict of Interest Statement

The authors declare that the research was conducted in the absence of any commercial or financial relationships that could be construed as a potential conflict of interest.
